# Manipulation of host immune defenses by effector proteins delivered from multiple secretion systems of *Salmonella* and its application in vaccine research

**DOI:** 10.3389/fimmu.2023.1152017

**Published:** 2023-04-04

**Authors:** Guodong Zhou, Yuying Zhao, Qifeng Ma, Quan Li, Shifeng Wang, Huoying Shi

**Affiliations:** ^1^ College of Veterinary Medicine, Yangzhou University, Yangzhou, Jiangsu, China; ^2^ Jiangsu Co-innovation Center for the Prevention and Control of Important Animal Infectious Diseases and Zoonoses, Yangzhou, China; ^3^ Department of Infectious Diseases and Immunology, College of Veterinary Medicine, University of Florida, Gainesville, FL, United States; ^4^ Joint International Research Laboratory of Agriculture and Agri-Product Safety, Yangzhou University (JIRLAAPS), Yangzhou, China

**Keywords:** host defenses, *Salmonella* infections, secretion systems, effector proteins, vaccine research

## Abstract

*Salmonella* is an important zoonotic bacterial species and hazardous for the health of human beings and livestock globally. Depending on the host, *Salmonella* can cause diseases ranging from gastroenteritis to life-threatening systemic infection. In this review, we discuss the effector proteins used by *Salmonella* to evade or manipulate four different levels of host immune defenses: commensal flora, intestinal epithelial-mucosal barrier, innate and adaptive immunity. At present, *Salmonella* has evolved a variety of strategies against host defense mechanisms, among which various effector proteins delivered by the secretory systems play a key role. During its passage through the digestive system, *Salmonella* has to face the intact intestinal epithelial barrier as well as competition with commensal flora. After invasion of host cells, *Salmonella* manipulates inflammatory pathways, ubiquitination and autophagy processes with the help of effector proteins. Finally, *Salmonella* evades the adaptive immune system by interfering the migration of dendritic cells and interacting with T and B lymphocytes. In conclusion, *Salmonella* can manipulate multiple aspects of host defense to promote its replication in the host.

## 1 Introduction

The ability of *Salmonella* to be transmitted through a fecal-oral route of infection makes it one of the major worldwide public health concerns (1, 2). The intestinal mucus layer provides a barrier against invasion of the epithelium by *Salmonella*. Commensal microflora in the intestinal mucosal can also promote intestinal stability and prevent pathogens from invading the intestine (3–5). Although the above mentioned barriers play an important role, but some *Salmonella* can also cross the epithelial barrier and reach the lamina propria where they encounter resident macrophages, dendritic cells (DCs), and intra-epithelial lymphocytes (6). One of their (e.g., resident macrophages, DCs, etc.) most important roles is to eliminate *Salmonella* by engulfing it and respiratory burst oxidase (RBO) and reactive oxygen species (ROS) (7). In addition, the ubiquitination modification is an enzymatic cascade reaction by which intracellular *Salmonella* can be labeled with (8). This is one of the pivotal “eat-me” signals that help to initiate the process of autophagy. Finally, ubiquitination can activate the process of inflammation, which also contributes to the resistance to intracellular *Salmonella* infection (9).

In addition to destroying invading *Salmonella*, phagocytes create a bridge between innate and acquired immunity by presenting antigens to T cells, thereby enabling the development of long-term immunity (10–12). It has also been suggested that cellular immune responses seem to be more effective in defending against *Salmonella* infection, and CD4^+^ Th1 cells have been shown to play a major role (13). This is probably due to the production of IFN-γ and TNF-α (hallmarks of Th1 cell response) that activate macrophages to kill intracellular pathogens (14). Previous studies have suggested that CD8^+^ T cells participate in secondary, but not primary, bacterial clearance. However, there is also evidence that the CD8^+^ CTL response plays an important role in resolving primary infection with attenuated *Salmonella* strain (15). Moreover, the production of anti-*Salmonella* IgG is essential to enhance phagocytosis in the adaptive immune response (16). In general, the T-cell mediated immune response is vital in host control of *Salmonella* infection, including primary and subsequent infection clearance (13, 15). Likewise, B cells are also crucial to the maintenance of a proper immune defense by antigen presentation and generation of protective antibodies (17). Thus, both cellular and humoral responses are likely key components of protective immunity.

Despite the presence of various antimicrobial mechanisms in the host, *Salmonella* have evolved various strategies to overcome these defense mechanisms (18). In Gram-negative bacteria, there are currently known six types of protein secretion systems, identified as type I to type VI secretion systems (T1SS-T6SS), each of which shows a considerable diversity (19, 20). To date, five secretion systems have been described in *Salmonella*, including the T1SS, T3SS, T4SS, T5SS and T6SS (19). After entering the intestine, *Salmonella* can use effector proteins secreted by the secretory systems to compete with intestinal flora and establish a colonization advantage (21). Eventually, the bacteria will be absorbed into the host cells, thus promoting the invasion of *Salmonella*. During this period, T3SS plays an extremely important role (22). Meanwhile, *Salmonella* resides within a host-derived membrane compartment, so-called *Salmonella*-containing vacuoles (SCVs) (23). The presence of *Salmonella* within SCVs avoids the killing effect of the cytoplasmic environment and contributes to its replication. The formation of SCVs can also reduce the presentation of antigenic peptides by DCs or other cell types, thus affecting the adaptive immune response and contributing to the establishment of systemic infection by *Salmonella* in the later stage (23).

With increasing antibiotic resistance of *Salmonella*, there is an urgent need to develop novel agents and efficient vaccines for the treatment and control of *Salmonella* (24). In recent years, it has been found that pathogenicity of bacteria is closely related to various virulence proteins or effector proteins secreted by their own, but these proteins are not essential components for bacterial survival (25). Therefore, the agent or vaccine targeted by this can not only play an essential role in the response to bacterial infection, but also produce less selective pressure on bacteria, thus reducing the possibility of bacterial resistance (26). In summary, the development of vaccines targeting different secretion systems during the *Salmonella* infection may be an effective strategy to block the spread of the disease.

In this review, we summarize the current knowledge of how pathogenic *Salmonella* utilize different secretion systems to modulate immune system and facilitate bacterial invasion and colonization. Herein, we describe the various vaccine candidates targeting the secretion systems, with a discussion on their advantages and disadvantages in the context of use scenarios. Through these aspects, we hope to provide a potential novel strategy that may be applied to the development of vaccines against *Salmonella*.

## 2 Manipulation of host defense by *Salmonella*

### 2.1 Breaking the intestinal barrier: The first step in *Salmonella* infection

**Figure 1:**
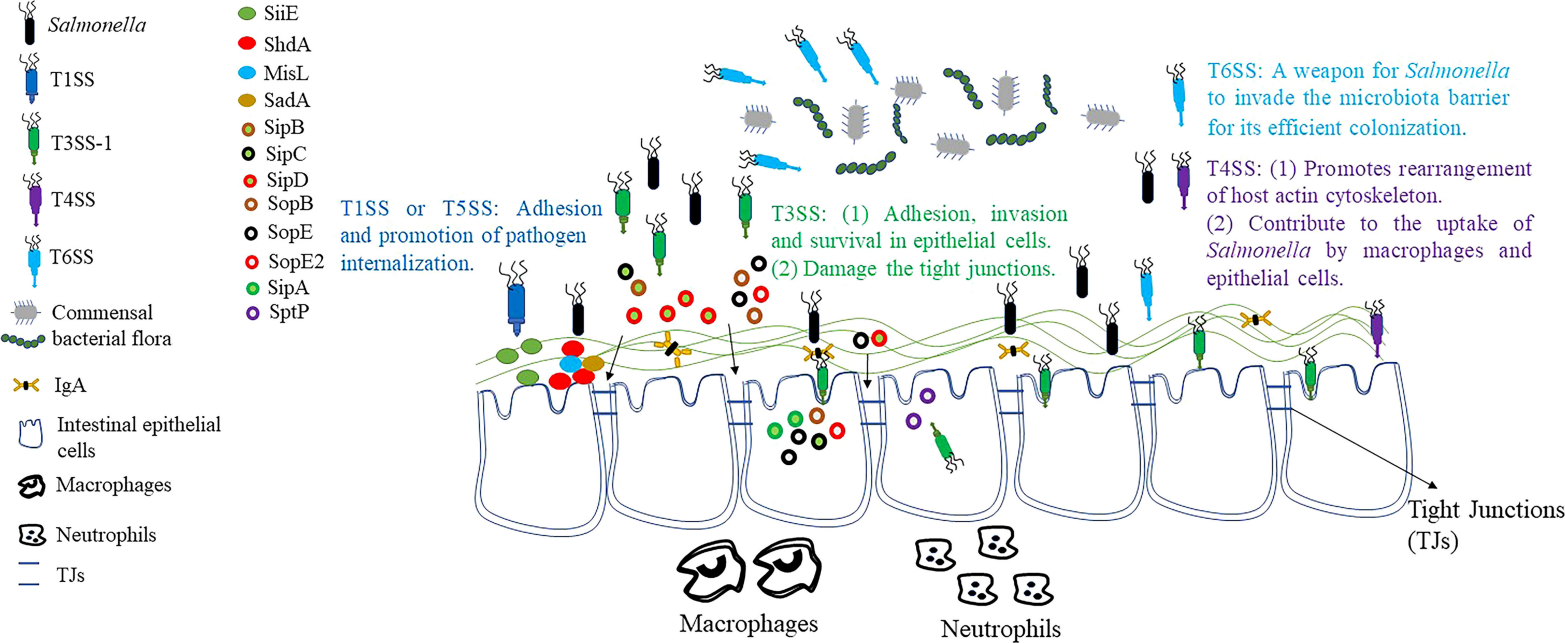
Breakthrough of the intestinal barrier by *Salmonella*. Before it reaches the intestine, *Salmonella* utilizes the T6SS to compete with intestinal commensal microorganisms. T1SS and T5SS facilitate the attachment and internalization of *Salmonella* to IECs. T4SS also contributes to host cytoskeletal rearrangement, which in turn promotes the uptake of *Salmonella* by macrophages and IECs. Through T3SS-1, *Salmonella* secretes effector proteins that lead to membrane ruffles, macropinocytosis and bacterial internalization, and ultimately invasion of host cells. At the same time, the effector protein secreted by T3SS-1 helps *Salmonella* to destroy TJs, which in turn breach the intestinal barrier. However, *Salmonella* also secretes effector proteins, such as SptP, that restore membrane structure after bacterial internalization and reverse the effects of membrane ruffles caused by other effector proteins.

The intestinal commensal flora plays a critical role in protecting the integrity of the intestinal mucosa (3). Among different secretion systems, T6SS is a contact-dependent secretion device capable of directly injecting effector proteins into other bacteria as well as eukaryotic cells (27). Many Gram-negative enteric pathogens, including *Vibrio cholerae*, *Pseudomonas aeruginosa* and *Bacteroides fragilis* can use its T6SS to defeat other bacteria (28). It suggests that T6SS may help to compete with intestinal flora for effective invasion of the intestinal mucosa. One such secretion system is also encoded in the *Salmonella* pathogenicity island 19 (SPI-19) present in serotypes Dublin, Weltevreden, Gallinarum and Enteritidis (29). The deletion of SPI-19 in these serotypes significantly affects the survival and colonization of *Salmonella* in cells and organs, and induces fast bacterial clearance. The SPI-19 deleted strain of *S*. Dublin also competed significantly weaker than the wild-type strain when co-cultured with strains of pathogenic *E*. *coli*, suggesting that this T6SS plays an important role in pathogenicity by killing commensal bacteria in the intestine (30, 31). *S*. Typhimurium harbors a T6SS encoded in SPI-6, which contributes to the capability of *Salmonella* to colonize mice (32). Subsequently, it was demonstrated that *S*. Typhimurium can also use SPI-6 T6SS to kill *Klebsiella oxytoca in vitro* (21). In a word, these results suggest that T6SS and its effector proteins may be a powerful weapon for *Salmonella* to compete with other intestinal flora as they breach the intestinal barrier (**Figure 1**).

After breaking through the blockade of the intestinal flora, the adhesion of *Salmonella* to intestinal epithelial cells (IECs) is a central step in the process of pathogenesis. The initial contact between *Salmonella* and polarized IECs is established by T1SS (33). This system secretes SiiE, a huge non-fimbrial adhesin that enables the bacterium to adhere the apical surface of host cell. BapA is encoded by SPI-9 and is also secreted by T1SS. It is required for *S*. Enteritidis to penetrate through IECs, constitutes a first stage in pathogenic processes, and is essential for *S*. Typhi to adhere to ECs (34, 35).

In addition to the adhesion proteins secreted by the above-mentioned T1SS, the proteins of the T5SS also play an important role in the intestinal invasion of *Salmonella* (35, 36). In *Salmonella*, three adhesins of the autotransporter protein family have been characterised earlier. ShdA and MisL are important monomeric adhesins while the putative adhesin SadA is a trimer autotransporter adhesins (TAA) (37–39). The ShdA is the only determining factor known to be required for persistence of *S*. Typhimurium in the mouse caecum and for efficient and extended shedding of the bacterial with the faeces (40). MisL is an autotransporter protein encoded by SPI-3. The *misL* mutant colonized poorly *in vivo* in comparison to the corresponding parental strain, with the bacterial loads recovered significantly lower than those of the wild-type *S*. Typhimurium strain SL1344 (37, 41). Moreover, expression of MisL enabled *S*. Typhimurium to bind fibronectin to its cell surface, resulting in adhesion to fibronectin-coated glass slides and in increased invasiveness for epithelial cells. These data indicate that MisL represents a potential extracellular matrix adhesin involved in intestinal colonization. Previous studies have shown that all members of the TAA family are adhesion proteins, and SadA is similar in structure to YadA protein (a member of the TAA family) of *Yersinia enterocolitica* (35). This result suggested that the SadA is probably also an important mediator of *Salmonella* adhesion. In addition, expression of SadA led to the agglutination of cell, formation of biofilm, and increased adhesion capability to human IECs (39). In conclusion, these results indicate that the colonization, adhesion and invasion of *Salmonella* in the intestine may require the cooperation of multiple secretory systems or multiple effector proteins (**Figure 1**).

Intercellular tight junctions (TJs) connect IECs to form a physical barrier that restricts bacterial pathogen invasion and migration (42). *Salmonella* has also developed various strategies to destroy TJs (**Figure 1**). Recent studies discovered that the T3SS-secreted effector proteins SopB, SopE, SopE2, and SipA are responsible for TJs structure and function disruption (43–45). In contrast, AvrA may play an important role in the stabilization of TJs. Previous studies have found that the presence of AvrA stabilizes intestinal TJs as well as normal cellular permeability, and the normal structure of TJs is more severely impaired by AvrA-deficient strains (46). To summarize, AvrA stabilized TJs despite the fact that the other T3SS effector proteins, SopB, SopE, SopE2, and SipA, are reported to disrupt TJs.

After successful adhesion to IECs, *Salmonella* can promote its internalization by different secretory systems or effector proteins (**Figure 1**). Currently, there are six effector proteins of *Salmonella* (SipA, SipC, SopB, SopE, SopE2 and SptP) that can regulate the actin cytoskeleton directly or indirectly (47, 48). Nucleation of microfilaments and their bundling by SipC leads to cytoskeletal rearrangements in cultured cells that result in membrane ruffles below the site of *Salmonella* attachment (49). Moreover, SipA promotes filament assembly and stabilizes filaments once they have formed (50). Thus, the combined activities of SipC and SipA promote the composition of actin filaments in proximity to attached *Salmonella*, and stabilize these filaments against disassembly by host regulatory proteins. In addition, the conversion of unbranched filaments into the branched filament networks that drive membrane evagination requires the stimulation of Rac (a member of the Rho family of GTPase, modulate the polymerization of actin) by SopE, as well as the downstream activation of the Arp2/3 (a crucial regulator of the dynamics of the actin cytoskeleton) complex (51). Finally, RhoG (a Rho family small GTPase implicated in cytoskeletal regulation) is indirectly activated by phosphoinositide phosphatase SopB, which aggravates the membrane ruffling phenomenon (52). SopE and SopE2 effector proteins can also lead to actin cytoskeletal rearrangement and pro-inflammatory cytokine expression by activating CDC42 (a member of the Rho family of small GTPases and a key regulator of the actin cytoskeleton) (53, 54). However, both Rac and CDC42 activation were subsequently down-regulated by GAP activity of another effector protein, SptP, which restored the membrane structure after bacterial internalization and reversed the membrane ruffling caused by other effector proteins (55, 56). In recent years, it has been found that SPI-1-deficient *S*. Typhimurium still has a residual invasion ability, which seems to depend on the outer membrane protein Rck (57, 58). Rck is able to bind to epidermal growth factor receptor (EGFR) and activate Arp2/3 through Rac1 and Akt, which leads to bacterial internalization through the zipper mechanism (58).

The T4SS is used by pathogenic microorganisms to transport macromolecules such as DNA, proteins, and toxins across the host cell. It has been found to be associated with a variety of pathogens, including *Legionella* spp., *Bartonella* spp., *Brucella* spp., *Coxiella* spp. and *Helicobacter pylori* (59, 60). For instance, the *Helicobacter pylori* Cag T4SS has an important role in the pathogenic mechanisms of gastric cancer and peptic ulcer disease (61). However, current studies have shown that only a few serotypes of *Salmonella* contain T4SS, and due to its particularity, there is little information about its role in *Salmonella* infection. Furthermore, a previous study found that *Salmonella* strains with the T4SS were more likely to enter and survive in ECs or macrophages than those without the T4SS (62). Therefore, T4SS may also play an important role in promoting the internalization of *Salmonella* (**Figure 1**).

### 2.2 Inflammation: A double-edged sword for *Salmonella* infection

**Figure 2:**
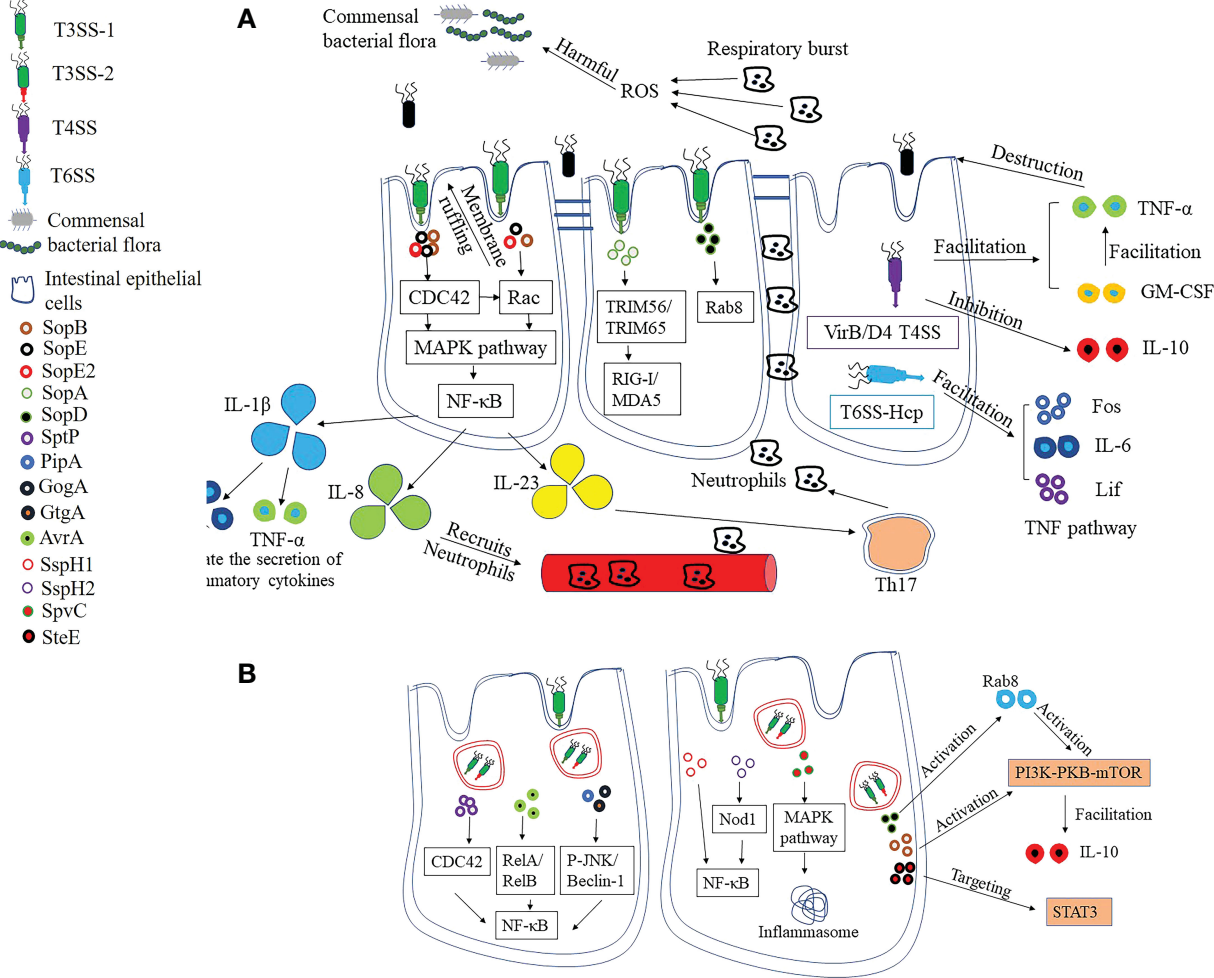
Pro-inflammatory and anti-inflammatory strategies of *Salmonella*. **(A)** Pro-inflammatory strategies. The effector proteins SopB, SopE and SopE2 secreted by T3SS are the central components of pro-inflammatory response, which leads to pro-inflammatory cytokine release by facilitating the activation of NF-κB. The production of pro-inflammatory cytokines recruits neutrophils, which contributes to the destruction of intestinal commensal flora and promotes *Salmonella* colonization. SopA and SopD, meanwhile, target innate immune inflammatory signals to stimulate inflammation and contribute to the amplification of the inflammatory response. T4SS-containing *Salmonella* can suppress the anti-inflammatory cytokine IL-10 secretion while increasing TNF-α and GM-CSF levels, resulting in intestinal epithelial barrier dysfunction and disruption of integrity. Hcp, one of the structural and effector proteins of T6SS, is involved in the regulation of TNF signaling pathways, including upregulation of Fos, IL-6 and Lif levels. **(B)** Anti-inflammatory strategies. The excessive inflammatory response leads to epithelial cell death, which in turn affects *Salmonella* survival. SptP, the effector protein of *Salmonella*, can inhibit inflammation by limiting the activity of CDC42. There are also some effector proteins that can affect NF-κB activation, which in turn reduces the pro-inflammatory response. In addition, the host mitogen-activated protein kinase (MAPK) is inactivated by SpvC, which also inhibits host autophagy and prevents the formation of inflammasome. SopB and SopD can stimulate the PI3K-PKB-mTOR signaling pathway, respectively, which inhibits the inflammatory response. Finally, the SteE (GogC) protein targets STAT3, a signaling pathway that restores homeostasis after an inflammatory response.

The pro-inflammatory response is a core factor in the pathogenicity of *Salmonella* (63). On the one hand, *S*. Typhimurium can initiate intestinal inflammatory responses through the stimulation of innate immune receptors by conserved bacterial products such as lipopolysaccharide (LPS), peptidoglycan or flagellin; on the other hand, it can also bypass these immune receptors to induce inflammation (63). The ability of *S*. typhimurium to stimulate inflammatory signaling is strictly dependent on SopB, SopE, and SopE2, and the absence of these three effector proteins fails to induce inflammatory signaling (64). Among them, SopE can activate Rac-1 and CDC42 independently, whereas SopE, SopB, and SopE2 can induce CDC42 release from ECs (56, 65). Rac-1 and CDC42 both belong to the Rho family of GTPases, which can lead to the activation of NF-κB and the release of pro-inflammatory cytokines such as IL-1β and IL-23 (66). Alternatively, SopA and SopD can target innate immune inflammatory signals to stimulate inflammation without binding to innate immune receptors, contributing to amplification of the inflammatory response (67, 68). It was also demonstrated that *Salmonella* can establish an advantage in competition with the commensal flora by activating the inflammatory responses. In conclusion, inflammation alters the balance between the intestinal commensal flora and *S*. Typhimurium in favor of *Salmonella* colonization in the host gut (**Figure 2A**).

In addition to helping *Salmonella* compete with normal intestinal flora, the change of pro-inflammatory cytokine profile also helps to change the permeability of ECs, thus promoting the invasion of *Salmonella* (**Figure 2A**). Currently, only a few serotypes of *Salmonella* have been reported to contain T4SS. Compared to strains without T4SS, VirB/D4 T4SS-containing *S*. *enterica* Serovar Heidelberg inhibits the secretion of the anti-inflammatory cytokine IL-10 when infecting IECs (69, 70). However, IL-10 deficiency has been linked to increased intestinal permeability, inflammation, and dysfunction, potentially contributing to the successful invasion and persistence of *Salmonella* in host cells. In the study, it was likewise demonstrated that infection of ECs by T4SS-containing *S*. *enterica* Serovar Heidelberg induced elevated levels of TNF-α and GM-CSF, and that changes in the expression of these cytokines may impair epithelial barrier function and thus contribute to bacterial invasion of IECs (69). Hcp has been proposed as a core component and hallmark secreted protein of T6SS, but little is known about the role of Hcp in infection. Expression of Hcp protein in BHK-21 cells by plasmid pEGFP-N1-hcp revealed that it is mainly localized in the cytoplasm and is involved in the regulation of TNF signaling pathways, including up-regulation of Fos, IL-6 and Lif levels, and downregulation of Ccl20, Ccl2 and Map3k8 (71). These findings suggest that T6SS may also contribute to the regulation of inflammation caused by *Salmonella*, which in turn changes the permeability of ECs.

An excessive inflammatory response will also lead to host cell apoptosis, which in turn puts pressure on the survival of *Salmonella*. As a result, *Salmonella* regulates the inflammatory response by expressing effectors that maintain host homeostasis (**Figure 2B**). Overall, effector proteins of *Salmonella* antagonize the onset of the inflammatory response through two mechanisms.

In the first mechanism, effector proteins directly antagonize signaling pathways triggered by agonists or pro-inflammatory effectors (63). SptP, for example, can effectively counteract the inflammatory response induced by SopE and SopE2, primarily by suppressing CDC42 activity (72). PipA, GogA, and GtgA were all able to cleave the transcription factors RelA (p65) and RelB of NF-κB, which in turn effectively limited the inflammatory response induced by *S*. Typhimurium (73). AvrA is also an important anti-inflammatory protein that inhibits the NF-κB pathway by suppressing P-JNK and Beclin-1 molecules (74, 75). SspH1 is mainly localized in the nucleus and down-regulates the production of pro-inflammatory factors by decreasing NF-κB-dependent gene expression (76). The deficiency of SspH2 had no effect on the virulence of *Salmonella enterica*, but reduced the colonization *in vivo* and promoted the expression of IL-1, TNF-α, IL-12 and iNOS cytokines, indicating that SspH2 is an important anti-inflammatory effector protein (77). Also, one study has shown that SspH2 is able to interact with Nod1 to regulate inflammation (78). Nod1, an intracellular receptor, is associated with the activation of NF-κB, suggesting that SspH2 exerts its effects also through the downregulation of NF-κB (79). Autophagy relies on the activation of inflammasome as part of the innate immune response and contributes to the host’s defense against *Salmonella* infection (80). It was shown that the effector protein SpvC of *Salmonella* can inhibit autophagy and reduce the levels of NLRP3 and NLRC4 (81). Finally, the presence of SpvC exacerbated systemic infection with *S*. Typhimurium by suppressing fever and intestinal inflammation in mice (82).

There are also some effector proteins that stimulate anti-inflammatory pathways to help maintain host homeostasis, which is the second mechanism by which *Salmonella* resists the host inflammatory response (**Figure 2B**). For example, in addition to stimulating the secretion of inflammatory cytokines, SopB activates the PI3K-PKB-mTOR signaling pathway and induces the production of the anti-inflammatory cytokine IL-10 to maintain host cell homeostasis (83). In addition, SopD can also exert anti-inflammatory effects by activating Rab8, which subsequently further stimulates the PI3K-PKB-mTOR signaling pathway (68). Finally, the SteE (GogC) effector protein acts on signal transducer and activator of transcription 3 (STAT3), a signaling pathway for homeostatic restoration after an inflammatory response (84).

### 2.3 Ubiquitination and deubiquitination: Identification and localization of *Salmonella*

**Figure 3:**
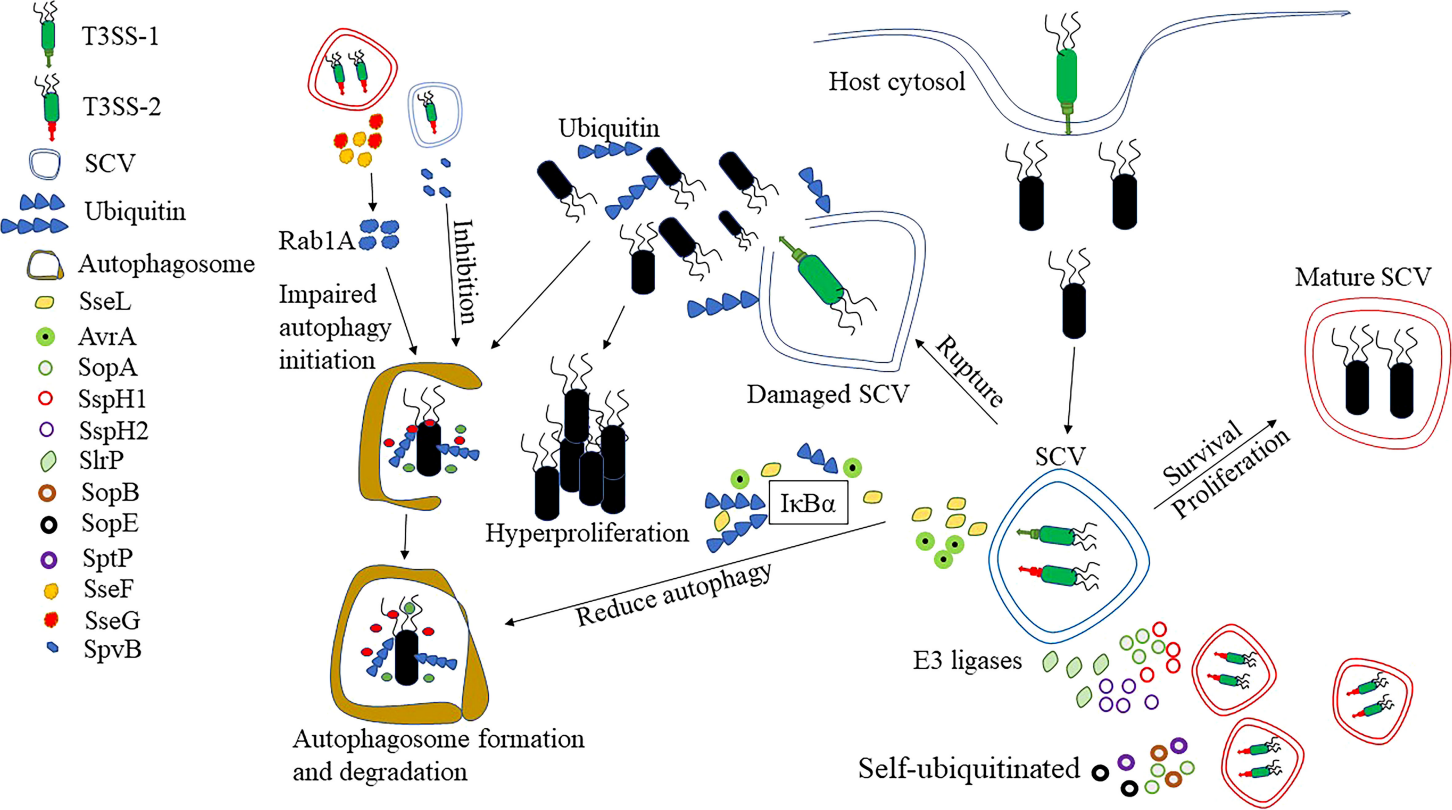
Response of *Salmonella* to ubiquitination and autophagy. After entering the cytosol, *Salmonella* can develop a unique ecological niche, SCVs. By the presence of SCVs, *Salmonella* can avoid recognition by ubiquitination, but studies have shown that the integrity of SCVs can be disrupted by T3SS-1. Some of the *Salmonellae* escape into the cytoplasm and hyper-proliferate, but there are also some bacteria recognized by ubiquitination. At the same time, ubiquitin also recognizes the damaged SCVs and the bacteria that remain inside the SCVs, and eventually clears the SCVs and bacteria by enzymatic reaction. However, *Salmonella* can effectively avoid the accumulation of ubiquitin aggregates and aggregate-like induced structures by secreting deubiquitinating enzymes, such as SseL and AvrA, which in turn prevent the recognition of ubiquitin-like modifications by autophagy receptors. There are also effector proteins, such as SopA, SspH1, SspH2 and Slrp that can act as E3 ligases to ubiquitinate host proteins and thus inhibit inflammatory responses. Finally, effector proteins such as SopA, SopB, SopE or SptP can also self-ubiquitinate to influence the antimicrobial response. The disruption of ubiquitination by SseL is also able to reduce the occurrence of autophagy. SseF and SseG, on the other hand, can interact with the small GTPase Rab1A in the host cell to inhibit autophagy initiation. Finally, SpvB depolymerizes actin, inhibiting host cell autophagy, which is also important for *Salmonella* intracellular survival.

The ubiquitin cascade reaction system is also able to regulate protein function, inflammation and immunity, ultimately affecting the innate and adaptive immune response to pathogens (85). Recognition by the ubiquitination system is an important tool for effective identification of intracellular *Salmonella*, but *Salmonella* can also evade the host immune response by targeting the ubiquitination pathway with secreted effector proteins (**Figure 3**).

SseL is T3SS effector protein with deubiquitinase activity that effectively avoids the accumulation of ubiquitin aggregates and aggresome-like induced structures, which in turn prevents the recognition of ubiquitin-like modifications by the autophagy receptor proteins (86). It was shown that SseL-deficient strains had significantly more ubiquitination-like aggregates around SCVs compared to wild-type *Salmonella*, while inducing the onset of autophagy. In addition, SseL affected the ubiquitination and degradation of IκBα, which in turn inhibited NF-κB activation. In contrast, SseL-deficient *Salmonella* induced a stronger inflammatory response, which may be associated with increased production of NF-κB-dependent cytokines (87). Therefore, as an important effector protein, SseL can further affect the occurrence of autophagy and inflammation through the regulation of ubiquitination modification. AvrA is also an effector protein with deubiquitinating enzyme activity, which effectively dissociates ubiquitin from IκBα and β-catenin (88). Moreover, the normal expression of AvrA in *Salmonella* helps stabilize IκBα and β-catenin during bacterial-host cell interactions, thereby inhibiting NF-κB signaling and suppressing inflammatory responses.

There is also a class of effector proteins in *Salmonella*, such as SopA, SspH1, SspH2 and Slrp, all E3 ubiquitin ligases, which are able to ubiquitinate protein substrates, and some are even able to self-ubiquitinate (**Figure 3**) (89). SopA was found to behave similarly to the mammalian HECT E3 ubiquitin ligase, preferentially recruiting three E2-coupled enzymes, UbcH5a, UbcH5c, and UbcH7E (90). Infection of HeLa cells with wild-type and SopA-deficient *Salmonella* revealed that the mutants were able to reduce the number of polymorphonuclear leukocytes (PMN) migrating *via* the epithelium, and thus it was hypothesized that SopA may be involved in regulating *Salmonella*-induced intestinal inflammation through ubiquitination of bacterial or host proteins. In addition, SspH1 can bind to PKN1 through its leucine-rich repeat (LRR) domain, allowing ubiquitination-proteasomal dependent degradation of PKN1 (91). It has been shown that PKN1 is a potent positive regulator of androgen receptor (AR) signal transduction (92). Also, previous studies have shown that AR can affect the number of neutrophils, macrophage activation and susceptibility to bacteria in mice (93, 94). Thus, the ubiquitination and degradation of PKN1 in cells by SspH1 further leads to the inhibition of AR, with possible effects on a number of cellular functions. As a member of the E3 ubiquitin ligase family, SspH2 can interact with NOD1 and NOD2 to regulate host innate immunity (95). In the study, SspH2 was found to specifically bind to NOD1 and induce ubiquitination modifications, leading to about four-fold higher NOD1 hyperactivation than the original. It also bound to NOD2 in the same way, resulting in a ten-fold increase in activation rate over basal activation. There was also an NF-κb-dependent elevation of IL-8 after SspH2 interacted with NOD1 and NOD2. All these results suggest that SspH2 super-activates NOD1 and NOD2, which in turn increases pro-inflammatory cytokine secretion. Slrp is also an important E3 ubiquitin ligase that binds to thioredoxin (involved in the control of many physiological processes and immune regulation) as well as mediates its ubiquitination, which results in a significant decrease in thioredoxin activity and increased cell death (96).

Another binding target of SlrP was shown to be ERdj3 of the Hsp40/DnaJ family, which plays an important role in the proper folding of proteins (97). However, the binding of SlrP to ERdj3 significantly reduced the interaction of ERdj3 with its substrate. It may lead to the accumulation of unfolded proteins in the endoplasmic reticulum, which eventually leads to cell death. Such an increase in host cell death caused by *Salmonella* effector proteins may, to some extent, contribute to the escape of bacteria from infected cells and is also necessary to infect new cells as well as to facilitate dissemination.

Finally, *Salmonella* is also capable of attaching ubiquitin to its own proteins using host E3 ligases, the more important of which are SopA, SopB, SopE or SptP (**Figure 3**) (89). It has been shown that both SopA and SopE can be degraded by the ubiquitination-proteasome pathway mediated by HsRMA1 (98, 99). And SptP, another representative of self-ubiquitination, can undergo proteasome-dependent degradation after being labeled by ubiquitination (89, 98). SopB, the T3SS effector protein of *S*. Typhimurium, can diversify its function by targeting to different cellular compartments in a ubiquitin-dependent manner (100). In contradiction to this, the activity of SopB is also down-regulated by ubiquitination mechanisms within the host cell, such as targeting it to lysosomal degradation (101). A further study showed that the E3 ubiquitin ligase TRAF6 is primarily responsible for SopB ubiquitination, which was totally prevented by TRAF6 absence (102). As the analysis of E2 showed, TRAF6-mediated ubiquitination of SopB requires UbcH5c rather than other E2-coupled enzymes *in vitro* and *in vivo*. As speculated by the above studies, the corresponding effector proteins minimize the impact on bacterial survival by self-ubiquitination to decrease the ubiquitinating enzymes in the host cells. At the same time, effector proteins are rapidly degraded after they exert their functions, preventing excessive accumulation, which may help to avoid adverse effects on hosts or pathogens.

### 2.4 Autophagy: An important weapon for intracellular *Salmonella* clearance

Autophagy is an essential component of the innate immune system which contributes to the intracellular clearance of *Salmonella* (103). Upon entrance into the IECs, *Salmonella* also induces the onset of autophagy. At present, it is believed that *Salmonella* can induce autophagy through two pathways (**Figure 3**) (80). The first pathway involves the recognition of *Salmonella* by ubiquitin (104). The intracellular *Salmonella* is generally thought to be present within the SCVs, but some can use their T3SS to impair the structural integrity of the SCVs, thereby escaping into the cytoplasm to achieve high replication rates (105). Intracellular *Salmonella* can be rapidly recognized by the host ubiquitination system, leading to the formation of an intensive layer of ubiquitin chains around the bacteria. Subsequently, ubiquitin-modified *Salmonella* is identified by autophagy adapters (e. g. NDP52, OPTN and p62). These adapters guide the bacteria to the primary autophagosome by binding to the ubiquitin-modified bacteria through their ubiquitin-binding domains and further interacting with the membrane anchoring protein LC3 of the autophagosome. The second pathway recognizes the damaged SCVs, and upon destruction by T3SS, the entire SCVs, including the *Salmonella* within it, is degraded by the autophagy pathway (104).

Similarly, *Salmonella* has also evolved various strategies to evade autophagy, in which T3SS and its secreted effector proteins play a crucial role (**Figure 3**). After infection of ECs, it was shown that the *Salmonella* mainly replicated in the SCVs and the ubiquitinated structures were also mainly surrounding the SCVs (106). This seems to be relevant to the effector proteins secreted by T3SS, as the formation of ubiquitinated structures is significantly reduced in cells infected with T3SS-deficient *Salmonella*. Both SseF and SseG of *S*. Typhimurium are secreted by T3SS, which impairs the activation of autophagy by directly interacting with Rab1A, a small GTPase in host cell (107). Upon recognition of *Salmonella* by the ubiquitination machinery, the autophagy receptor p62 is capable of recruiting LC3 that promotes autophagosome formation (106, 108). A co-localization of p62 and LC3 was also found in the ubiquitinated structures induced by *Salmonella*-infected cells. The deficiency of SseL significantly increased the number of ubiquitinated structures, p62 and LC3, compared to cells infected with wild-type bacteria (86). It was shown that autophagy may be further hindered by SseL through the reduction of ubiquitinated structures around SCVs and the presence of autophagy markers p62 and LC3. Moreover, autophagosome formation was found to be increased in SpvB mutant strains compared to wild-type *Salmonella* (109). In addition, it was shown that the infection of zebrafish by SpvB mutant strain resulted in increased expression of LC3 and Beclin1, and double membrane-like autophagosome structure also observed, indicating that SpvB can inhibit autophagic activity (110, 111). The polymerization of the actin backbone is involved in the formation of autophagy, and the SpvB effector protein secreted by *S*. Typhimurium depolymerizes host cell actin (112). Therefore, it is speculated that SpvB may inhibit host cell autophagy by depolymerizing actin.

### 2.5 Reducing the persistence of IgG^+^ plasma cells in BM: A disruption of the humoral immune response

Antibodies facilitate the uptake of bacteria by phagocytes to prevent infection, and ultimately the destruction of internalized bacteria by phagocytes (16, 113). There are two ways in which antibody exerts its antimicrobial effect. The first one is that pathogens recognized by Fc receptors on macrophages, which is called opsonophagocytosis (113). Alternatively, antibodies binding to the surface of the pathogen can activate the proteins of the complement system (114). The activation of the complement system leads to opsonophagocytosis by binding to complement receptors on phagocytes (115). In addition, other complement elements will recruit phagocytes to the site of infection, and the terminal of complement can directly lyse microorganisms by forming pores in their membranes. However, *Salmonella* can also disrupt the humoral immune response with the help of the secretory system (**Table 1**). Bone marrow (BM) is the central tissue for hematopoiesis and immune memory, and serum IgG is mainly produced by IgG-secreting plasma cells in the BM. It has been shown that SiiE, secreted by the *Salmonella* T1SS, can reduce the persistence of IgG^+^ plasma cells in the BM to prevent effective humoral immune memory (116). This is the only effector protein that has been reported to interfere with the adaptive immune response in the T1SS.

**Table 1:** Effector proteins involved in adaptive immune response.

Secretion system	Component/Effector	Functions in daptive immune response	References
T1SS	SiiE	Reduced the persistence of IgG^+^ plasma cells in BM	(116)
T3SS-1	SopB	Reduces the level of negatively charged lipids on the surface of SCV, dissociates endocytic transport proteins and avoids degradation by lysosomes	(117)
	SopF	Maintaining the stability of the SCV	(118)
	SipA	(1) Facilitate the fusion of early phagosomes with SCV (2) Avoids the maturation of phagosomes to lysosomes	(82, 119)
T3SS-2	SifA	(1) Inhibition of DCs migration (2) Essential for SCV stability and SIFs formation	(120, 121)
	SopD2	Prevents the entry of Rab32 to SCV, which in turn interrupts endocytosis	(122, 123)
	SseJ	Esterifies cholesterol, dissociates cholesterol esters, and promotes SCV stability	(124, 125)
	SseF	(1) Inhibition of DCs migration (2) Anchoring of SCV around the Golgi network	(126, 127)
	SseG	Anchoring of SCV around the Golgi network	
	SseI	Inhibition of migration of macrophages and DCs	(128)
	SteD	(1) Promotes the ubiquitination and surface exhaustion of mMHCII, which in turn decreases T-cell activation (2) Reduces co-stimulatory molecules on the surface of antigen-presenting cells, such as CD86 and CD97	(129–131)
T3SS-1/T3SS-2	GtgE	(1) Manipulation of SCV transport and prevention of Rab29/32 accumulation in SCV (2) Prevention of fusion of SCV with lysosomes, contributing to SCV stabilization	(132, 133)
	PipB2	(1) Promotes the stability of SCV (2) Promotes the extension of SIFs, which in turn contributes to material and nutrient supplies (2) Inhibits the migration of DCs	(134, 135)
	SteA	(1) Control of membrane dynamics (2) Related to the formation of SIFs	(136)
	SspH2	Inhibits the migration of DCs	(134)
	SlrP	Inhibits the migration of DCs	

### 2.6 Intervention of DCs migration and antigen presentation: Blocking the activation of cellular immune response

The activation of CD4^+^ T cells can effectively target the infection of *Salmonella*, therefore, if *Salmonella* wants to establish a systemic infection, it must interfere with the normal biological function of DCs (**Table 1**). The effector protein secreted by T3SS appears to be more important for the survival of *Salmonella* inside DCs (**Table 1**). It was shown that the effector protein SseI secreted by T3SS inhibits normal cell migration of primary macrophages and DCs *in vitro*, and also inhibits migration of DCs to the mouse spleen *in vivo* (128). In addition, the researcher found that effector proteins such as SseF, SifA, SspH2, SlrP, and PipB2 seem also to participate in the inhibition of DCs migration, but the exact mechanism needs to be further investigated (134). These evidences suggest that *Salmonella* can interfere with the normal function of DCs by secreting effector proteins, which in turn impairs the initiation of host adaptive immune response.

Bacterial growth can be hindered by the cytoplasm of ECs and phagocytes. Therefore, the cytosolic environment represents an early selective pressure on *Salmonella* (23, 137). For survival, the majority of *Salmonella* is present in a specialized niche SCVs after uptake by cells (23). The presence of SCVs effectively prevents cellular killing of the bacteria and reduces antigen presentation of DCs or other antigen-presenting cells (APCs), which is in turn affects the adaptive immune response. SCVs, on the other hand, frequently binds to lysosomes, causing SCVs rupture and exposing the bacteria to an environment rich in hydrolytic enzymes even if they remain intact. But *Salmonella* has evolved various ways to both restrain lysosomal binding and maintain SCVs integrity (**Table 1**), in which SifA plays an important role (120, 121). The absence of the effector protein SifA is capable of causing more than 50% of the SCVs to rupture, leading to the release of the bacteria into the cytosol, which is detrimental to the survival of the bacteria. The cysteine protease GtgE, also secreted by T3SS-1 and T3SS-2, is able to manipulate SCVs transport and prevent the accumulation of Rab29 on SCVs (132). Also, GtgE is able to cleave Rab32 and prevent the fusion of SCVs with lysosomes, contributing to the stabilization of the SCVs membrane (133). In the same way, SopD2 prevents the accumulation of Rab32 to SCVs, thus hindering the endocytosis of host cells (122, 123).

There are also numerous effector proteins secreted by *Salmonella* to regulate the lipid and protein content of SCVs, which will contribute to the stability of SCVs (**Table 1**) (138). For example, the SopB effector protein, secreted by T3SS-1, is able to reduce the level of negatively charged lipids on the surface of SCVs, which subsequently leads to the dissociation of many endocytic transporter proteins from SCVs and avoids the occurrence of degradation by lysosomes (117). SopF is a phosphatidylinositol-binding effector protein that binds to a variety of phosphatidylinositols in the protein-lipid overlays after delivery by *Salmonella* and, furthermore, knockout of the *sopF* leads to increased cleavage of SCVs, suggesting that it may promote the stability of SCVs (118). SipA, an actin binding protein, is required for effective entrance of *Salmonella* into host cells, where it can recruit Synaxin8 instead of the host R-SNARE molecule, promote early phagosome fusion with SCVs, avoid maturation of phagosomes to lysosomes, and promote pathogen survival (119, 139). Furthermore, SseJ dissociates cholesterol esters from the phospholipid bilayer in SCVs membranes, which is necessary because increased cholesterol content can affect membrane fluidity, signaling, sorting, and transport (124). In conclusion, as an essential effector protein, SseJ plays an important role in maintaining the stability of SCVs. It was shown that SCVs formed by SseF or SseG-deficient *Salmonella* undergo irregular movement in the cytoplasm (126). Hence, the prevalence of these two effector proteins in multiple serotypes provides strong evidence that they may contribute to bacterial growth in host cells. *Salmonella*-induced filaments (SIFs) are a device for nutrient uptake by bacteria within the SCVs. PipB2, an effector protein secreted by both T3SS-1 and T3SS-2, plays an important role in SCVs formation and is also required for the extension of SIFs (135). In combination with SifA, this effector protein serves to facilitate membrane exchange and nutrient delivery by allowing the formation of tubules from SCVs and extension along the microtubule cytoskeleton. SteA also plays an important role in controlling membrane dynamics, and the absence of SteA reduces the ability of *Salmonella* to form SIFs, increases the aggregation of SCVs, as well as the formation of abnormal vacuoles (136). In conclusion, the presence of SCVs not only contributes to bacterial survival, but also avoids the activation of the adaptive immune response, especially the cellular immune response.

The uptake of bacteria, after processing by DCs, results in the presentation of antigenic peptides to CD4^+^ T cells *via* MHC II molecules (140). MHC II molecules play a key role in adaptive immunity by displaying antigenic peptides on the surface of APCs (e.g., DCs) to CD4-restricted T cells, leading to their activation, proliferation and differentiation (141). The infection of DCs by *Salmonella* exhausts mature MHC II molecules (mMHC II) on the cell surface. A recent study has shown that SteD acts as an important effector protein that can direct the E3 ligase MARCH8 to binding with mMHC II which results in mMHC II ubiquitination and surface exhaustion, ultimately reducing the activation of T cells (129) (**Table 1**). In addition, inhibition of T-cell activation by SteD was accomplished by reducing the levels of at least three proteins (including MHC II, CD86, and CD97) on the surface of antigen-presenting cells (130, 131). Among them, CD97 mainly stabilizes the immune synapse between DCs and T cells. After degradation by SteD, it eventually inhibits DCs-T cell interactions and reduces T cell activation. Thus, SteD suppresses T-cell immunity through two distinct processes.

## 3 Vaccine studies related to *Salmonella* secretion systems

### 3.1 Subunit vaccine

T3SS-1 is required for IECs invasion and barrier penetration, and it is an extracellular needle-like device required for effector protein injection into host cells (142). PrgI and SipD are both essential components of the T3SS-1 tip complex (143). Because they are common and highly conserved among all virulent *Salmonella* species, they may be ideal candidate targets for a broad-spectrum vaccination against *Salmonella* infection. The levels of immunogenicity induced after immunization of mice with PrgI and SipD proteins alone or in combination by different immunization routes (subcutaneous, intranasal and oral) were investigated and showed that high levels of IgG and IgA titers against both proteins could be induced, where the levels of SipD-specific antibodies were higher (**Table 2**) (144). In the same study, it was also shown in protective studies that immunization with SipD protein alone or in combination with PrgI protected mice from the lethal challenge by *S*. Typhimurium with 100×LD_50_. Furthermore, a study showed that administering SipD protein through intranasal or intragastric routes induced strong IgG (in all immune pathways) and IgA (in intranasal and oral immune pathways) antibody responses and protected mice from lethal challenge by *S*. Typhimurium or the *Shigella* spp (**Table 2**) (145). This is mostly because the structural proteins that constitute T3SS are shared by all pathogenic *Salmonella* and *Shigella* spp., particularly the tip protein. Regardless of *Salmonella* serotype, the two types of T3SS are used to interact with host cells, particularly the tip protein and the first translocation effector protein of *Salmonella*, both of which are essential for pathogenicity. Based on this, previous vaccine studies fused the tip protein SipD of T3SS-1 to the first translocation effector protein SipB, named S1 protein, and fused the tip protein SseB of T3SS-2 to the first translocation effector protein SseC, named S2 protein, then vaccinated the mice with S1 and S2 alone or in combination (**Table 2**) (146). Following that, challenge with *S*. Typhimurium or *S*. Enteritidis resulted in a 60% survival rate regardless of serotype, indicating that fusion with tip and translocator proteins is a feasible vaccine candidate. However, none of the candidate vaccines elicited an effective mucosal immune response, which may be connected to the immunological route of the protein, as studies have shown that the parenteral route is not a very efficient approach to stimulate IgA production (161–163).

**Table 2:** Application of effector proteins in vaccine development.

Vaccine types	Protein targets	Functions	Evaluation indicatos	References
Subunit vaccines	PrgI/SipD	The T3SS needle tip protein	(1) Antibody titers (2) Survival rates	(144)
	SipD		(1) Antibody titers (2) Survival rates	(145)
	SipB/SipD/ SseB/SseC	The T3SS needle tip protein and the first translocator	(1) Antibody titers (2) Antibody-secreting cells (3) Survival rates (4) Cecal inflammation	(146)
Live vaccines	SptP	T3SS effector protein and mediating alterations in the actin cytoskeleton of host cells	(1) Antibody titers (2) Lymphocyte proliferation assay (3) Serum cytokine analyses (4) Survival rates	(147)
	SopB	T3SS effector protein and affects cytoskeletal rearrangement, host-cell invasion and chloride homeostasis	(1) Antibody titers and IgG isotype analyses (2) Serum cytokine analyses (3) Stimulation of memory T-cell response (4) Survival rates	(148)
	SsaV (with AroC)	Part of the structural gene of the T3SS-2 secretory apparatus	(1) Antibody titers (2) IgA enzyme-linked immunospot assay (3) Lymphocyte proliferation (4) IFN-γ assays	(149–153)
	SsaV (with Fur)		(1) Histopathological Evaluation (2) FACS Analysis for T-cell Population (3) Antibody titers	(154)
	SsaV (with Hha)		(1) Histopathological Evaluation (2) T-cell activation (3) Antibody titers	(155)
	SifA (with AroC)	(1) Inhibition of dendritic cell migration (2) SCV stability and SIF formation	(1) *In vitro* antigen presentation assays (2) Splenocyte interferon-γ ELIspot analysis (3) The levels of CTL induction by *Salmonella* (4) Impact on dendritic cell maturation (5) Levels of proinflammatory cytokines	(156)
Inflammasome-targeted vaccines	SspH2	(1) *Salmonella* T3SS-2 effector (2) Inhibition of DC cell migration	(1) Levels of proinflammatory cytokines, LDH, and caspase-1 activation (2) Levels of ROS, NO, [Ca^2+^]i and MMP (3) The activation and differentiation of T lymphocytes in mouse spleens (4) Coagulation assay (5) Survival rates	(157, 158)
Effector proteins act as secretory signals	SptP	(1) T3SS effector protein and mediating alterations in the actin cytoskeleton of host cells (2) Inhibits inflammation (3) Self-ubiquitination	(1) The ability of *Salmonella* to present antigens to MHC Class I pathway (2) The levels of CTL induction by *Salmonella* (3) Survival rates	(159)
	SseJ	T3SS effector protein and esterifies cholesterol	(1) The frequency of survivin-specific IFN-γ secreting cells (2) Infiltration with CD8 T cells (3) Tumor growth and survival rates	(160)

### 3.2 Live vaccines with deficient function of effector proteins

SptP, secreted by T3SS-1, regulates the dynamics of the cytosolic actin backbone and plays an important role in *Salmonella* invasion (53, 55). C50336 Δ*sptP*, a *sptP*-deficient strain, was inoculated in mice, and the humoral and cellular immune responses of the immunized mice were studied afterwards, revealing that the vaccine strain was highly immunogenic and provided 100% protection against *S*. Enteritidis after challenge (147). It indicates that the deletion of *sptP* may be a new target for the development of salmonellosis vaccine. Subsequently, live attenuated vaccine strains were constructed by introducing *sptP* mutations in different *S*. Enteritidis strains and the protective efficacy was investigated in chickens (164). The results showed that a strong cellular immune response was induced by both lymphocyte proliferation and cytokine assays, and the level of specific IgG antibodies in the immune group was significantly increased, demonstrating the high immunogenicity of the live vaccine. After challenge, it was also able to reduce clinical signs and pathological changes in chickens, with a highest protection rate of 100%. In summary, *sptP*-deficient *Salmonella* strains may have good potential for application in both mammals and avian species (**Table 2**).

By invading through the mucosa and colonizing lymphoid tissues, *Salmonella* is able to induce a strong mucosal and cellular immune response in the host persistently (165). Based on this, the attenuated *Salmonella* is often used as a vector to deliver protective antigens of other pathogens (148, 166–168). However, inappropriate attenuation of *Salmonella* vectors often leads to a severe inflammatory response, which is unacceptable (169). SopB, secreted by T3SS-1, is capable of exacerbating the inflammatory response induced by *Salmonella* (148). It was shown that *sopB* deficiency in *Salmonella* impairs the ability to elicit local inflammatory responses and fluid secretion into the intestinal lumen, but also enhances the immunogenicity of *Salmonella* as a vector for the presentation of exogenous antigens (148, 168). Subsequently, elevated immunogenicity was demonstrated by the delivery of the *Streptococcus pneumoniae* surface protein PspA through *Salmonella*, indicating that the deletion of the SopB contributes to the development of live attenuated vaccines (**Table 2**). Based on this, our laboratory introduced the *sopB* deficiency into the vectors of *S*. Choleraesuis, which we expected to further improve the immunogenicity of vector vaccine. As a result, immunization of the mouse model provided a better protection against either *Streptococcus suis*, *Mycoplasma hyopneumoniae* or porcine circovirus infection (166–168).

SsaV is a necessary component of the T3SS-2 secretion apparatus, and the secretion of various effector proteins into the host must be initiated by sensing the neutral pH of the host cytoplasm, and SsaV is the key protein of this transition switch (**Table 2**) (170). Oral immunization of adult volunteers with *S.* Typhi or *S*. Typhimurium (both deficient in *aroC* and *ssaV*) showed that ZH9 (candidate strain of *S*. Typhi) not only had a better security, but also elicited high titers of antibody responses (149). In contrast, immunization of volunteers with WT05 (candidate strain of *S*. Typhimurium) also elicited high titers of antibody response, but it was shedding in the feces until 23 d. In adult volunteers, the immunogenicity of M01ZH09 (ZH9) immunization with or without carbonate buffer solution was compared separately, demonstrating a well-tolerated with or without carbonate buffer, a mild adverse event after vaccination, and no fever or long-term shedding (150). Furthermore, the vaccine was immunogenic, with more than 88% or 93% of participants in both groups having IgA antibody-secreting cells detectable by ELISPOT, and 81% of participants in both groups producing LPS-specific IgG on day 14. Lymphocyte proliferation and IFN-γ production also showed that the vaccine elicited a strong cellular immune response. A subsequent clinical trial was conducted to determine the tolerability and immunogenicity of a single dose of M01ZH09 (ZH9), which showed that adverse effects were less frequent, the time of fecal shedding was reduced, and the immune response was dose dependent, with the highest dose (5 × 10^9^ CFU) being the most immunogenic (151). Previous studies in healthy adults demonstrated the immunogenicity and acceptable safety of ZH9, 151 children were subsequently recruited in Vietnam in the study, of whom 101 subjects were orally immunized with a single dose of M01ZH09 (ZH9) (152). The results showed that high titers of LPS-specific IgA and IgG could be detected in the serum after immunization, and although no bacteremia was observed, some children experienced adverse reactions, indicating that the vaccine was appropriately immunogenic, but safety should be improved.

The absence of SsaV influences the secretion of effector proteins. However, immunization of mice with the SsaV-deficient *Salmonella* still elicited O antigen-specific immune responses and improved survival of mice after challenge (171). However, the introduction of the mentioned strains into immunocompromised mice still caused lethal infections, indicating that further attenuation is necessary (**Table 2**) (171). Therefore, one study has introduced an additional *fur* deletion into SsaV-deficient *Salmonella*, a gene that contributes to acid tolerance and iron acquisition (154). The double-deletion strain is safe in immunocompromised mice, while being sufficiently immunogenic to enhance protection against *Salmonella*. Hha, a nucleoid-associated protein, is able to downregulate the expression of some virulence and invasion-related genes. The introduction of Hha mutation in SsaV-deficient *Salmonella* has reduced the systemic colonization ability and the adverse effects on immunocompromised hosts (155). Moreover, humoral and cellular immune responses were enhanced after the introduction of Hha mutation. These results suggest that the combination of SsaV-deficient *Salmonella* with the Hha mutation is a live attenuated candidate vaccine that can be safely used in immunocompromised hosts.

An attractive aspect of live vector vaccines is their ability to stimulate a robust cellular immune response and the efficient delivery of antigen to the MHC I presentation pathway, which is particularly important for CD8^+^ T cell development (172). It is generally accepted that *Salmonella* is preferentially present in SCVs upon entry into the cell, and if the escape of *Salmonella* from SCVs into the cytoplasm can be increased, it may facilitate antigen delivery *via* the MHC I pathway, thereby enhancing the CTL response (**Table 2**) (23). Studies have shown that SifA has an important role in maintaining the integrity of SCVs in macrophages and ECs (120). In one study, after the deletion of *sifA* in AroC-deficient *Salmonella*, most of the *Salmonella* was shown to successfully escape into the cytoplasm compared to the AroC-deficient *Salmonella* only, however, no subsequent increase in MHC I presentation efficiency to the model antigen (Ovalbumin) was detected, nor was an increase in cytotoxic T cell or IFN-γ production levels (156). Another study, however, has shown that the *ssaV* and *aroC* double deletion strains induced accelerated maturation of DCs, higher production of TNF-α, IL-12, and IL-1 cytokines, and, most importantly, more efficient antigen presentation than the *sifA* and *aroC* deletion strains (153). The above results suggest that enabling the escape of *Salmonella* from SCVs may not be sufficient to enhance the MHC I presentation efficiency of the antigen. In contrast, the increased efficiency of antigen presentation after *ssaV* deficiency demonstrates that further screening is needed to investigate whether other effector proteins are involved in interfering with antigen presentation, which may contribute to the development of more effective vaccines targeting CTL.

### 3.3 Inflammasome-targeted vaccines

Activation of the inflammasome contributes to the clearance of intracellular bacteria (**Table 2**). To construct an effective vaccine targeting the inflammasome, the C-terminus of an *E. coli* EscI protein was fused to the N-terminal of SspH2, and attenuated *Salmonella* was used for delivery (157). The strain fused to the C terminus of EscI protein significantly increased IL-1 and IL-18 secretion and cell pyroptosis in mouse intraperitoneal macrophages, while causing less colonization in organs and fewer pathological changes in the spleen and liver than the strain delivered with only the N terminus of SspH2. The fusion protein SspH2-EscI was shown to translocate into macrophages and activate NLRC4 inflammasome, which limits the colonization of *Salmonella* in the spleen and liver. Subsequently, the vaccine potential of strains delivering the fusion protein SspH2-EscI was investigated, and the results showed that compared to strains delivering only the SspH2 N-terminal or empty plasmids, the colonization of organs was effectively reduced after challenge and the survival rate of mice was improved, indicating that recombinant *Salmonella* expressing the SspH2-EscI fusion protein enhanced the activation of caspase-1 in macrophages and protected mice from *Salmonella* challenge (158).

### 3.4 Effector protein as a secretory signal

As mentioned previously, attenuated *Salmonella* strains are an effective vector for delivery of immunogenic proteins of other pathogens (158, 166–168). However, after entering the cells, *Salmonella* is mainly present in the SCVs, where antigens are mainly presented through MHC II molecules and induce mainly a CD4^+^ T cell immune response (23, 173). However, CTL induced by CD8^+^ T cells plays an important role in both viral infections and tumor-associated diseases, so it is also extremely important how *Salmonella* can be used to elicit a higher CTL response (174, 175). In addition to defects in SCVs stability-related genes that allow *Salmonella* to escape from SCVs to the cytoplasm, some researchers have also fused heterologous antigens to effector proteins secreted by the T3SS so that the antigens can be delivered to the host cell cytoplasm *via* the T3SS (**Table 2**). Previous research has shown that fusion expression of MHC I epitopes of influenza virus nucleoproteins embedded inside SptP proteins can successfully translocate them into the host cytoplasm and induce a CTL response capable of dissolving influenza virus-infected cells (159). As CTL plays a major role in the fight against lymphocytic choroid plexus meningitis virus (LCMV) of mice, the immunogenic protein of LCMV was subsequently embedded inside SptP (159). Oral immunization protected mice from lethal challenge by LCMV, demonstrating that the use of the secreted protein of *Salmonella* T3SS as a secretory signal can successfully deliver protective antigens of LCMV as well as provoke an effective immune response. It was discovered that delivery of tumor-associated antigens (TAAs) using the above strategy was also extremely effective for the induction of CD8^+^ T cells. In a study, over 20 effector proteins of SPI-2 were fused with TAAs separately, and it was discovered that fusion of TAAs with SseJ and replacement of the *sseJ* promoter with that of *sifB* effectively induced CD8^+^ T cells with strong antitumor activity (160). It demonstrated that the effector proteins of *Salmonella* as secretory signals for antigen delivery may be effective cancer vaccine platforms.

## 4 Summary and outlook

It is generally accepted that physical barriers and immune responses are beneficial for host defense against infection because they limit the replication and transmission of pathogens. However, the presence of the immune system is a double-edged sword for *Salmonella*; on the one hand they limit the replication and systemic spread of *Salmonella*, and on the other hand, *Salmonella* can utilize the immune system to compete with the normal flora and establish a systemic infection. The manipulation of *Salmonella* to these defense mechanisms is mostly related to the secretory systems and the effector proteins presented by these systems. In recent years, the function of some of the effector proteins delivered by the secretory system has been successfully characterized, and their functional analysis has allowed a better understanding of the pathogenesis of *Salmonella* and deciphering the potential mechanisms by which *Salmonella* evades the host immune system. However, there are many aspects of secretory systems and effector proteins that are still unknown. It also makes the prevention and control of Salmonella infection difficult because the mechanisms of immune escape are ill-defined. Although the use of antibiotics can effectively control the spread of *Salmonella*, appearance of multidrug-resistant bacteria is an additional serious threat to public health. Vaccine programs are the most effective and cost-effective measure to prevent and control pathogenic infections. As a zoonosis with numerous serotypes, current vaccines provide protection against only a limited number of serotypes of *Salmonella*. The presence of T3SS in numerous serotypes makes it an ideal vaccine and drug target, although it is not clear which effector protein induces better protection against *Salmonella*. Therefore, further studies are necessary to investigate whether the combination of multiple proteins can provide better protection, especially to different *Salmonella* serotypes, in addition to investigating the vaccine potential of individual effector proteins. Other secretory systems also play an important role in adhesion and invasion, but only a few studies have explored these secretory systems and little is known about their role in the pathogenesis of *Salmonella*. Therefore, the possibility of various secretory systems as targets for *Salmonella* vaccine development should also be the focus in subsequent studies.

## Author contributions

HS, SW, and QL designed, supervised, and critically revised the manuscript. GZ drafted the manuscript. GZ, QM, and YZ did the reference collection. All authors contributed to the article and approved the submitted version.
